# Effectiveness of different socket dressing materials on the postoperative pain following tooth extraction: a randomized control trial

**DOI:** 10.25122/jml-2022-0140

**Published:** 2022-08

**Authors:** Ahmad Salem Assari, Hamad Saud Alrafie, Abdullah Homoud Al Ghashim, Faisal Nabeel Talic, Ahmed Mushabbab Alahmari, Meshal Yousef Al Manea, Rakan Yousef Alrashdan

**Affiliations:** 1 Department of Oral and Maxillofacial Surgery and Diagnostic Sciences, Riyadh Elm University, Riyadh, Saudi Arabia; 2 Riyadh Third Health Cluster, Saudi Ministry of Health, Riyadh, Riyadh, Saudi Arabia; 3 Riyadh Elm University, Riyadh, Saudi Arabia; 4 King Faisal Specialist Hospital and Research Centre, Riyadh, Saudi Arabia

**Keywords:** dry socket, post-extraction pain, Alvogyl, Cutanplast

## Abstract

We aimed to prospectively evaluate and compare the effectiveness of Alvogyl and Cutanplast as intra-alveolar dressings for managing pain associated with extraction and incidence of dry socket. All patients who underwent maxillary and mandibular teeth extraction and fulfilled our inclusion and exclusion criteria from Feb 2021 to Oct 2021 were included in our study. Patients who were diagnosed with postoperative pain after tooth extraction were randomly allocated to three groups: Group A (Alvogyl), Group B (Cutanplast), and Group C (placebo). Pain relief and healing of the socket were compared between these groups. The collected data were analyzed using the Chi-square test and Z test of proportionality. Alvogyl was superior to the other medication for providing initial pain relief, and the incidence of dry socket was significantly lower than in the Cutanplast and placebo groups (p<0.05). However, wound healing was statistically non-significant among groups A, B, and C (p>0.05). Alvogyl is still the material of choice in terms of pain relief, wound healing, and low incidence of dry socket. Furthermore, no statistically significant difference was detected between the groups in the biographic information, location, and condition of the extracted tooth, presence of a radiologic pathology, or type of extraction procedure. Moreover, whether it is the first extraction or not, Alvogyl and Cutanpast are comparable in postoperative pain management as intra-alveolar dressing materials.

## INTRODUCTION

Dry socket is a common post-extraction issue following the extraction of permanent teeth, particularly mandibular third molars [1]. Dry socket is also known as alveolar osteitis (AO), fibrinolytic alveolitis, alveolitis, alveolitis sicca dolorosa, localized osteitis, localised AO, septic socket, necrotic socket, and alveolalgia [2, 3]. For routine dental extractions, the incidence of dry socket ranges from 0.5 to 5.5 percent, and it can reach 38 percent in the case of surgical extraction of impacted mandibular third molars. On the third post-extraction day, patients typically complain of intense, radiating pain in and around the extraction socket, as well as halitosis; the socket is devoid of an organized blood clot and is filled with debris, revealing the underlying bone [3].

The inconsistency in the documentation of aetiology, risk factors, prevention, and treatment modalities represent a serious clinical problem. Dry socket is thought to have a multifactorial aetiology, with general factors like age and gender, decreased body resistance from systemic disease, nutritional deficiency, as well as local factors like anatomical location, traumatic surgery, smoking, clot fibrinolysis, local circulation, local anaesthesia, and vasoconstrictors [4–6]. Several studies [7] found a relationship between contraceptives and dry sockets. Contraceptives contain estrogen, which is thought to affect the coagulation system [8].

Bacteria have long been suspected of playing a role in the development of dry sockets in people with poor oral hygiene and pre-existing infections [2]. In a comprehensive study, Nitzan stated that anaerobic microorganisms play a role in the aetiology of dry sockets. Many investigations indicate that Treponema denticola, an organism typically linked with gingival disease, has significant extracellular plasmin-like activity [9]. Razanis *et al*. [10] showed the association between Actinomyces viscosus and *Streptococcus mutans* and this condition in animal models by demonstrating delayed socket repair following organism injection.

Many researchers tried to find a suitable technique for preventing dry sockets, the most common post-extraction issue. Systemic antibiotics, topical antibiotics, chlorhexidine, para-hydroxybenzoic acid, tranexamic acid, polylactic acid, steroids, eugenol-containing dressings, lavage, 9-aminoacridine, and others have been proposed to help avoid dry socket. However, there is still disagreement because no technique has gained widespread approval [11].

The therapy of dry sockets is as disputed as its cause and prevention. Any “treatment” advice is likely deceptive because the illness cannot be treated until the cause is identified. According to Fazakerlev and Field [12], the primary goal of dry socket therapy is to reduce pain until normal healing can commence, and in most cases, local remedies are sufficient. In some circumstances, systemic analgesics or antibiotics may be required. The use of intra-alveolar dressing materials is often suggested in the literature, despite the widely held belief that dressings inhibit extraction socket restoration. Although several treatments and carrier systems are commercially available, there is little scientific evidence to guide their use [11].

AO treatment is typically centered on soothing the patient and providing immediate pain relief until natural healing occurs. Alvogyl is a palliative drug that contains analgesic and anti-inflammatory Eugenol, antibacterial iodoform, and butamben (anesthetic) [13].

Absorbable gelatin sponges contain hemostatic material and can be utilized in surgical procedures where typical hemostasis is difficult to establish. The use of Alvogyl or an absorbable gelatin sponge (Cutanplast) can minimize postoperative pain severity, painkiller consumption, postoperative hemorrhage, and wound re-epithelialization [14]. Aside from its hemostatic properties, an absorbable gelatin sponge can be employed as a drug reservoir to enable prolonged drug release [15].

A prospective randomized experiment was necessary to objectively examine the relative efficacy of various dressings for the management of pain and promotion of healing in AO. The purpose of this study was to examine socket healing, the incidence of acute alveolar osteitis (AO), and related discomfort in people who had direct intra-alveolar Alvogyl injections *versus* those who received Cutanplast after molar tooth extraction.

## Material and Methods

A prospective randomized clinical trial single-blind was conducted on eighty-four patients in Riyadh Elm university hospitals and private clinics from February 2021 to October 2021.

### Methodology for Data Collection

All patients who reported to the department for dental extraction from February 1, 2021, to October 31, 2021, were included in the study after receiving approval from the research and ethics council. Pregnancy, radiation history, chemotherapy, hematologic diseases, bone diseases, acute infections or cystic lesions, allergies to Lidocaine, Ibuprofen, Alvogyl, and gelatin, and concomitant cellulitis/facial space infections were all considered exclusion factors. Traumatic extractions with fragmented alveolar bone, extractions requiring bone reduction, and extractions lasting more than 30 minutes were also excluded. In the standardized proforma created to collect the numerous criteria essential for accomplishing the study's objectives, all patients provided a complete case history.

Extractions were performed by a general practitioner and a dental intern. All participants' information about the objectives of the study and consent forms were signed. All the required information was documented in the questionnaire paper regarding name, age, gender, nationality, date, file number, mobile number, smoking, medical history, tooth number indicated for extraction, time of tooth extraction, and whether it was a first-time extraction. Preoperative clinical tooth evaluation (sound tooth for orthodontic purposes, badly decayed tooth, or remaining roots) and radiographic tooth evaluation (normal periapical area, periapical radiolucency, or radiopacity). Postoperative assessment of tooth extraction according to extraction procedure: simple extraction (with elevator and forceps only) or surgical extraction including flap and suture (with or without root separation, bone removal) and according to time consumed during extraction (within 30 min, within 1h, more than 1h).

Teeth extractions were carried out using 2% Lidocaine and 1:100,000 epinephrine. Infiltration was used to anesthetize the upper molars, while a combination of inferior alveolar nerve block and buccal infiltration was used to anesthetize the lower molars. Elevators and forceps were used for simple extractions, while a surgical handpiece and burs with standard saline irrigation were used for root separations.

Patients who complained of pain following tooth extraction were assessed to determine the source of the problem. A blinded assessor made a clinical diagnosis of AO based on the following characteristics: pain in and around the extraction socket that worsens with time, with or without radiation, between 1 and 3 days following the extraction.

To receive therapy for their ailment, patients were randomly assigned to one of three groups: A, B, or C, using a randomization table. Within these groups, patients were treated as follows:

- Group A: Alveogyl^®^ was used on extraction sockets manufactured by Septodont India Pvt. Ltd. 15.8 gm iodoform, 13.7 gm eugenol B.P., and 25.7 gm butamben).
- Group B: In the extraction sockets, a Cutanplast^®^ 10×10×10 mm gelatin sponge (MasciaBrunelli, Italy) soaked in 2% Lidocaine with 1:100,000 epinephrine was placed.
- Group C: Extraction sockets received no medication and acted as a control group.

### Procedure

A warm sterile saline solution was used to irrigate the socket. Curettage was avoided at all costs. Loose debris was removed with special care not to dislodge any remaining clots in the socket.

#### Alveogyl® Placing Technique

With the use of a sterile device, a few fibers of Alvogyl were put deep into the socket, ensuring that the denuded bone was entirely covered, followed by the installation of sterile gauze. After 5 minutes, the gauze was removed.

#### Cutanplast placement technique

Inside the socket, a single roll of Cutanplast was put in, followed by a sterile gauze piece to cover the socket. After 5 minutes, the gauze was removed.

Postoperative instructions were given to all participants. In addition, a prescription of Ibuprofen 400 mg (Prof, Tabuk Pharmaceuticals, Tabuk, Saudi Arabia) every 8 hours for the first 3 days was also given to the patients.

The participants recorded their pain intensity on the Wong-Baker Visual Analogue Scale (VAS) (0–10). A score of 0 indicates no pain, while a score of 10 indicates severe pain. Pain intensity was recorded after 3, 6, 12, and 24 hours of tooth extraction and on the second and third day. At each follow-up appointment, the dressings were assessed by a blinded assessor.

### Statistical Analysis

The data was assessed using the SPSS software package (Version 20). Descriptive statistics such as frequency distribution, mean, standard deviation, and percentages were analyzed. Analysis of pain statistics amongst the three groups was done using One Way Analysis of Variance. Post hoc analysis was done using Tukey's HSD. The proportions of dry socket incidence and healing signs at the follow-up periods compared to baseline were tested using the Chi-Square test and the Z test of proportionality, respectively. In all tests, a P value of less than 0.05 was considered significant.

## Results

**Figure 1:**
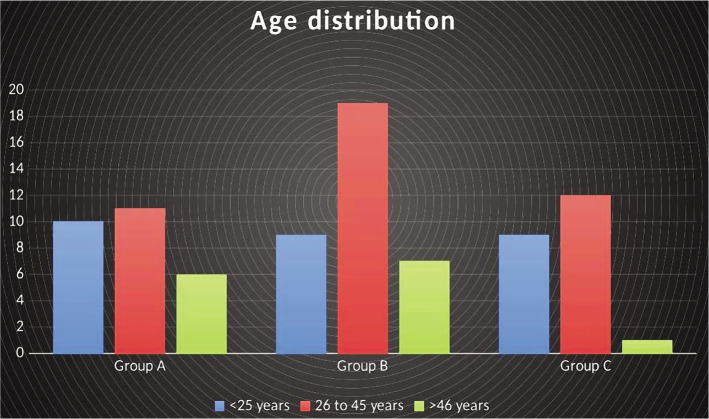
The age distribution of patients.

**Figure 2:**
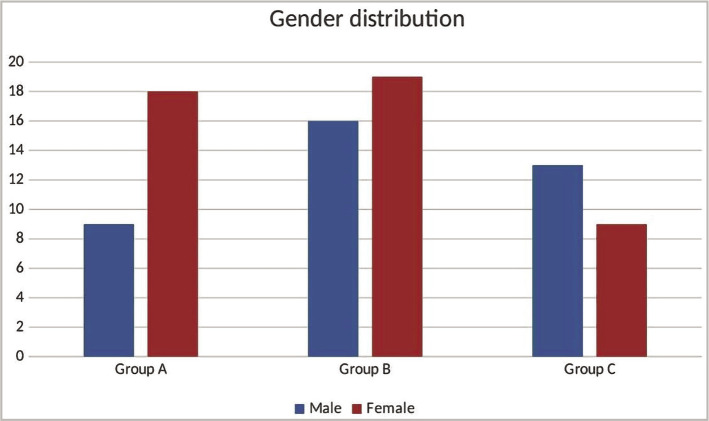
The gender distribution of the extraction.

In this study, a total of 84 subjects participated. These subjects were allocated to Group A (N=27), Group B (N=35), and Group C (N=22). The majority of participants were between 26 and 45 years old (Figure 1). Most of the participants in Group A and Group B were female, while in Group C, most were male (Figure 2). More than 80% of participants in Group A and Group B reported being non-smokers, while in Group C, nearly 41% reported being smokers. The proportion of first extraction participants was 3.7% in Group A, 11,4% in Group B, and 27.3% in Group C (27.3%). More than 50% of participants in Group A and Group B underwent upper tooth extraction, and in Group C, most underwent lower tooth extraction (59.1%). In Groups A and B, most extracted teeth were severely decayed. In Group C, an equal proportion of badly decayed teeth and remaining roots were extracted. The surgical extraction procedures increased from 3.7% in Group A to 5.7% in Group B (5.7%) and 18.2% in Group C. There were no statistically significant differences between the groups in any variable comparisons (Table 1).

**Table 1:** Comparison of study variables between the three intervention groups.

		Group A		Group B		Group C		P-value
		N	%	N	%	N	%	
**Age**	<25 years	10	37.0%	9	25.7%	9	40.9%	0.348
	26 to 45 years	11	40.7%	19	54.3%	12	54.5%	
	>46 years	6	22.2%	7	20.0%	1	4.5%	
**Gender**	Male	9	33.3%	16	45.7%	13	59.1%	0.197
	Female	18	66.7%	19	54.3%	9	40.9%	
**Smoking**	No	23	85.2%	28	80.0%	13	59.1%	0.081
	Yes	4	14.8%	7	20.0%	9	40.9%	
**First extraction**	No	26	96.3%	31	88.6%	16	72.7%	0.048*
	Yes	1	3.7%	4	11.4%	6	27.3%	
**Tooth**	Upper teeth	15	55.6%	19	54.3%	9	40.9%	0.530
	Lower teeth	12	44.4%	16	45.7%	13	59.1%	
**Tooth condition**	Sound (ortho)	1	3.7%	4	11.4%	2	9.1%	0.258
	Badly decayed	20	74.1%	23	65.7%	10	45.5%	
	Remaining roots	6	22.2%	8	22.9%	10	45.5%	
**Extraction procedure**	Simple	26	96.3%	33	94.3%	18	81.8%	0.145
	Surgical	1	3.7%	2	5.7%	4	18.2%	
**Time Taken**	Up to 30 mins	20	74.1%	29	82.9%	14	63.6%	0.295
	Up to 1 hour	4	14.8%	4	11.4%	7	31.8%	
	More than 1 hour	3	11.1%	2	5.7%	1	4.5%	

Chi-square test; * – Statistical significance at p≤0.05.

Dry sockets were present in 48.1% of cases in Group A, 63% of cases in Group B, and 91% of cases in Group C. A comparison of the three groups showed that the difference in proportions was statistically significant, indicating a significantly higher incidence of dry socket in Group C compared to Groups A and B (p=0.006).

The mean time for initial pain relief in Group A was 6.75±1.43 minutes, 10.20±2.78 minutes in Group B, and 22.34±3.03 minutes in Group C. These differences in mean values amongst the three groups were statistically significant, indicating significantly lower initial pain relief time in group A and significantly higher initial pain relief time in group C. Concerning time for complete resolution of pain, the mean was lowest in group A at 4.85±2.07 days, higher in group B at 7.35±2.96 days and highest in group C at 9.54±3.42. These differences in mean values amongst the three groups were statistically significant, indicating significantly low complete pain resolution time in group A and significantly high complete pain relief time in group C. Thus, overall pain management was better in Group A (Table 2).

**Table 2:** Pain relief with various medicaments.

	Group	N	Dry Socket Present	Mean & SD	ANOVA P-value	Post Hoc Analysis	
**Mean time for initial pain relief (Mins)**	A	27	13 (48.1%)	6.75±1.43	P<0.001, S	A vs. B	P<0.001, S
	B	35	22 (63%)	10.20±2.78		A vs. C	P=0.003, S
	C	22	20 (91%)	22.34±3.03		B vs. C	P<0.001, S
**Mean time for complete resolution of pain (Days)**	A	27	13 (48.1%)	4.85±2.07	P<0.001, S	A vs. B	P=0.002, S
	B	35	22 (63%)	7.35±2.96		A vs. C	P<0.001, S
	C	22	20 (91%)	9.54±3.42		B vs. C	P=0.016, S

S – Significant.

In group A, at baseline, there were 6 cases of empty sockets, which reduced to 4 on the third day, 3 on the fifth day, 1 on the seventh day, and no empty socket cases on the 10^th^, indicating complete healing. The reductions in empty socket cases from baseline to the third day, the fifth day, the seventh day, and the tenth day were statistically significant. Concerning exposed bone, at baseline, there were 6 cases, which were reduced to 3 on the third day, 2 on the fifth day, 1 on the seventh day, and no empty socket cases on the tenth, indicating complete healing. The reductions in exposed bone cases from baseline to the 3^rd^ day, the 5^th^ day, the 7^th^ day, and the 10^th^ day were statistically significant. Concerning redness around the socket, at baseline, there were 5 cases, which were reduced to 3 on the third day, 2 on the fifth day, 1 on the seventh day, and no redness around the socket on the tenth, indicating complete healing. The reductions in redness cases from baseline to the third, fifth, seventh, and tenth day were statistically significant (Table 3).

**Table 3:** Signs of healing (seen as the resolution of signs).

Groups	Dry socket signs		Follow up periods				
			Baseline	3^rd^ Day	5^th^ Day	7^th^ Day	10^th^ Day
**A**	Empty socket	No of cases (% of baseline)	6	4 (66.7%)	3 (50%)	1 (16.7%)	0 (0%)
		P-value (Baseline vs.Follow Up)	-	0.044, S	0.035, S	0.007, S	P<0.001, S
	Exposed bone	No of cases (% of baseline)	6	3 (50%)	2 (33.3%)	1 (16.7%)	0 (0%)
		P-value (Baseline vs. Follow Up)	-	0.035, S	0.021, S	0.007, S	P<0.001, S
	Redness around socket	No of cases (% of baseline)	5	3 (60%)	2 (40%)	1 (20%)	0 (0%)
		P-value (Baseline vs. Follow Up)	-	0.039, S	0.027, S	0.010, S	P<0.001, S
**B**	Empty socket	No of cases (% of baseline)	10	8 (80%)	5 (50%)	3 (30%)	2 (20%)
		P-value (Baseline vs. Follow Up)	-	0.134	0.043, S	0.018, S	P=0.002, S
	Exposed bone	No of cases (% of baseline)	9	6 (66.7%)	4 (44.4%)	3 (33.3%)	2 (22.2%)
		P-value (Baseline vs. Follow Up)	-	0.40, S	0.031, S	0.024, S	P=0.004, S
	Redness around socket	No of cases (% of baseline)	8	5 (62.5%)	4 (50%)	3 (37.5%)	2 (25%)
		P-value (Baseline vs. Follow Up)	-	0.046, S	0.043, S	0.033, S	P=0.003, S
**C**	Empty socket	No of cases (% of baseline)	11	9 (81.8%)	8 (72.7%)	6 (54.5%)	5 (45.4%)
		P-value (Baseline vs. Follow Up)	-	0.229	0.062	0.041, S	0.033, S
	Exposed bone	No of cases (% of baseline)	10	9 (90%)	8 (80%)	7 (70%)	5 (50%)
		P-value (Baseline vs. Follow Up)	-	0.484	0.344	0.057	0.035, S
	Redness around socket	No of cases (% of baseline)	9	7 (77.8%)	6 (66.6%)	4 (44.4%)	3 (33.3%)
		P-value (Baseline vs. Follow Up)	-	0.147	0.048, S	0.034, S	0.023, S

S – Significant.

In group B, at baseline, there were 10 cases of empty sockets, which were reduced to 8 cases on the 3^rd^ day, 5 cases on the 5^th^ day, 3 cases on the 7^th^ day, and 2 empty socket cases on the 10^th^ day. It was not statistically significant that empty socket cases dropped from baseline to the third day. However, the drop was statistically significant from baseline to the fifth day, the seventh day, and the tenth day. Concerning exposed bone, at baseline, there were 9 cases, which were reduced to 6 on the third day, 4 on the fifth day, 3 on the seventh day, and 2 on the 10^th^. The reductions in exposed bone cases from baseline to the 3^rd^ day, the 5^th^ day, the 7^th^ day, and the 10^th^ day were statistically significant. Concerning redness around the socket, there were 8 cases at baseline, which were reduced to 5 on the third day, 4 on the fifth day, 3 on the seventh day, and 2 on the 10^th^. The reductions in redness cases from baseline to the third, fifth, seventh, and 10^th^ day were statistically significant (Table 3).

In group C, there were 11 cases of empty sockets at baseline, which were reduced to 9 cases on the 3^rd^ day, 8 cases on the 5^th^ day, 6 cases on the 7^th^ day, and 5 cases on the 10^th^ day. The reductions in empty socket cases from baseline to the third day and from baseline to the fifth day were not statistically significant. However, the reductions were statistically significant from baseline to the 7^th^ day and 10^th^ day. Concerning exposed bone, there were 10 cases at baseline, which was reduced to 9 cases on the third day, 8 on the fifth day, 6 on the seventh day, and 5 on the 10^th^. The reductions in exposed bone cases from baseline to the third day, fifth day, and seventh day were not statistically significant. However, the reduction from baseline to the tenth day was statistically significant. At baseline, there were 9 cases, which were reduced to 7 on the third day, 6 on the fifth day, 4 on the seventh day, and 3 on the 10^th^. The reductions in redness cases from baseline to the third day were not statistically significant. However, from baseline to the 3^rd^ day, the 5^th^ day, the 7^th^ day, and the 10^th^ day were statistically significant.

When comparing groups concerning all three signs of the empty socket, exposed bone, and redness, Group A showed better signs of healing compared to groups B and C. Delays in healing were seen in Group C, which is different from groups A and B (Table 3).

## Discussion

Dry socket is a serious clinical problem that must be addressed. Following tooth extraction by both ordinary and specialized dentists, it is still the most common condition. In standard practice, a dry socket may not always be avoided entirely. The incidence varies between 1% and 4%, according to Archer [16] and MacGregor [17]. In this study, dry socket was reported to occur 1.64% of the time. The incidence of mandibular third molar extractions was between 16.8 and 31.2% by Milhon *et al*. [18] and Quinely *et al*. [19]. However, this was much less in the current study (11.38%). Patient age, gender, surgical technique, smoking, medical status, operator experience, and use of oral contraceptives may all play a role in the reported prevalence of dry socket.

In the current study, the most consistent clinical finding was discomfort, documented in all 84 sockets (100%). Larsen [20], Nusair and Abu Younis [21], and Nusair and Abu Younis [22] all made comparable observations. Furthermore, Nusair and Abu Younis [21] discovered an empty socket in every case and bare bone in 70% of them, but Larsen [20] puts these figures at 58 and 48%, respectively. However, the new study contradicts this traditional presentation of these two findings, finding unfilled sockets in only 32.14% of patients and barebone in 16.32%. The most common clinical finding among patients, according to the findings of this inquiry, was pain. As a result, the term “dry socket” may be inappropriate, and the phrases “post-extraction alvolalgia” or “post-extraction alveolitis” are preferable. As a result, the dry socket appears to be a kind of post-extraction alveolitis, in which the clot disintegrates partially or completely.

The capacity to effectively control pain is a critical skill in dental practice [22]. For both the patient and the doctor, pain management improves clinical outcomes and has far-reaching repercussions [23]. NSAIDs have considerably reduced tooth pain in multiple randomized, double-blind, placebo-controlled clinical trials and systemic investigations. Furthermore, these studies support the use of NSAIDs as the first line of treatment for dental pain [22, 24–26].

The use of intra-alveolar dressing materials for postoperative pain control is recommended [13]. Alvogyl is a dressing made up of several distinct components. Among the ingredients are Eugenol, sodium lauryl sulfate, calcium carbonate, and excipients. According to the manufacturer, Alvogyl is advised to treat post-extraction pain during complicated or traumatic surgeries.

The participants were mainly between the ages of 26 and 45, with an average age of 35.50. Other investigations produced similar results [27]. In this study, there was no statistically significant association between age and postoperative pain. Some research, such as Faizel *et al*. [28], established a statistically significant link between age group and dry socket, with the highest occurrence of dry socket in the 21–40 year age group.

The female-to-male ratio in this study was 1.2:1, with 45 (54%) of the 84 patients being female and 38 (46%) being male (Figure 2). Other studies found a modest female predominance in postoperative discomfort and dry socket, with a female to male ratio of 1.4:1 [17]. In this study, maxillary teeth accounted for 51% of the removed teeth, whereas mandibular teeth accounted for 49% (Figure 2). There was no statistical significance regarding the extraction site. On the other hand, some investigations discovered that the mandible is more prone to postoperative pain and dry socket [27, 28].

Females have a higher incidence of dry sockets than males, according to a study by MacGregor [17], which is consistent with our findings. This could be because oral contraceptives were not commonly used before 1960 [2]. According to Ygge *et al*. [29] and Sweet and Butler [19], oral contraceptive tablets increase fibrinolytic activity in the blood and saliva of women during the menstrual phase. The prevalence of AO was significantly higher among contraceptive users in our study.

Despite delayed healing in this study, groups A and B had uneventful healing after the seventh day compared to group C. Another study by Supe NB *et al*. [30] discovered that certain patients might experience delayed healing. On the tenth day, the Alvogyl group reported two cases of delayed healing, whereas the ZOE group reported nine cases of delayed healing, according to the study. Soukaina T. *et al*. [31] conducted another clinical study on Alvogyl and found a greater frequency of both alveolar osteitis and local (operative site) infection. When Alvogyl is connected to the extraction sockets, it may provoke a foreign-body giant cell reaction. The reaction makes the healing process more difficult.

In circumstances where more postoperative discomfort is expected, Alvogyl is indicated [31]. Furthermore, another study found that the wound healing of these sockets is histologically delayed. An immediate inflammatory infiltration, multiple foreign bodies in the Alvogyl-filled extraction socket, an inability to create a connective tissue scar, and persistent granulation tissue were all seen on histological examination [32]. However, in another study, the biocompatibility of the gelatin sponge (Cutanplast) was assessed, and it was established that the Cutanplast standard is a biocompatible material that causes platelet adhesion and aids in hemostasis without being cytotoxic or genotoxic [33]. Another randomized controlled study found that using Cutanplast following impacted third molar extraction surgery reduced edema significantly [34].

The results of this study revealed a statistically significant difference in postoperative pain control between Alvegy and Cutanpast. In a study by Ehab, Karim, and colleagues [14], there were no statistically significant variations in VAS pain levels between the two groups, namely Alvogyl and absorbable gelatin sponge as palatal surgical dressing materials.

Nusair and Abu Younis [21] and Amaratunga and Senaratne [35] found it most common in the third and fourth decades of life. According to Krogh [36], there was a major reduction in the following decades. The bulk of surgical extractions was done on patients in their third and fourth decades in these studies. Surgical extractions have been connected to dry socket syndrome [16, 18]. The results of the present investigation back up the previous findings.

For controlling postoperative pain, several intra-alveolar dressings and laser therapies were investigated. During the 7-day treatment period, no statistically significant difference in VAS pain scores was seen between patients treated with Alvogyl (Eugenol) and SaliCept (acemannan), demonstrating that SaliCept is a potent palliative treatment for dry socket [37]. According to the study, low-level laser therapy (LLLT) exhibits the fastest drop in VAS values. Although the Alvogyl and SaliCept groups experienced general pain relief by the sixth day after treatment, the LLLT group reported no pain by the third day [37].

To our knowledge, no comparative study between Alvogyl and Cutanplast has been published. Subsequently, multiple publications in the literature extolling the benefits of individual drugs can be bewildering as a result. A comparative analysis is essential to make an informed decision. In this study, the efficacy of drugs was determined by their ability to provide pain relief and clinical evidence of socket healing.

There were statistically significant differences in pain scores between Alvogyl and Cutanplast patients. When comparing Alvogyl to Cutanplast, Alvogyl had a faster onset of analgesia. Alvogyl was also able to provide pain relief that was both rapid and long-lasting. Additionally, Alvogyl required fewer dressing changes than the others.

According to an examination of the improvement in signs of dry socket after dressings with the two drugs under consideration, the signs of an empty socket and exposed bare bone in the Alvogyl treatment group completely disappeared by the seventh day. After the seventh day, just one (3.7%) socket in this group experienced persistent redness surrounding the afflicted area. On the other hand, two sockets in group B remained empty after the seventh day. In group B, two sockets had exposed bone more than seven days after treatment began. Redness around the socket remained for more than seven days in two Cutanplast-treated sockets and one Alvogyl-treated socket.

These findings show a statistically significant difference in results between the two groups, with Alvogyl showing higher pain relief and healing capability, as evidenced by clinical signs of improvement. Clinical criteria, on the other hand, cannot be used to evaluate the healing feature. Histologic validation is therefore essential.

One of the limitations of the study is the small sample size and that there are not enough surgical extraction instances. However, larger sample size is needed to verify that there is no statistically significant difference in postoperative pain between Alvogyl and Cutanpast. Patients with systemic disorders, acute infections, and cystic lesions should also be included in future studies.

## Conclusion

The present study concludes that there is a statistically significant difference between using Alvogyl or Cutanpast in postoperative pain management. Furthermore, no statistically significant difference was detected between the groups in the biographic information, location, and condition of the extracted tooth; the presence of a radiologic pathology, type of extraction procedure; and whether it was the first extraction or not Alvogyl and Cutanpast are comparable in postoperative pain management as intra-alveolar dressing materials.

## Data Availability

Further data is available from the corresponding author on reasonable request.
